# Anti-Inflammatory Effects of 6-Methylcoumarin in LPS-Stimulated RAW 264.7 Macrophages via Regulation of MAPK and NF-κB Signaling Pathways

**DOI:** 10.3390/molecules26175351

**Published:** 2021-09-02

**Authors:** Jin-Kyu Kang, You-Chul Chung, Chang-Gu Hyun

**Affiliations:** Jeju Inside Agency & Cosmetic Science Center, Department of Chemistry and Cosmetics, Jeju National University, Jeju 63243, Korea; wlsrbtjsrb@naver.com (J.-K.K.); jyc8385@hanmail.net (Y.-C.C.)

**Keywords:** 6-methylcoumarin, macrophage, inflammation, NF-κB, MAPK, coumarin

## Abstract

Persistent inflammatory reactions promote mucosal damage and cause dysfunction, such as pain, swelling, seizures, and fever. Therefore, in this study, in order to explore the anti-inflammatory effect of 6-methylcoumarin (6-MC) and suggest its availability, macrophages were stimulated with lipopolysaccharide (LPS) to conduct an in vitro experiment. The effects of 6-MC on the production and levels of pro-inflammatory cytokines (interleukin (IL)-1β, IL-6, tumor necrosis factor (TNF)-α) and inflammatory mediators (nitric oxide (NO), prostaglandin E_2_ (PGE_2_)) in LPS-stimulated RAW 264.7 cells were examined. The results showed that 6-MC reduced the levels of NO and PGE_2_ without being cytotoxic. In addition, it was demonstrated that the increase in the expression of pro-inflammatory cytokines caused by LPS stimulation, was decreased in a concentration-dependent manner with 6-MC treatment. Moreover, Western blot results showed that the protein levels of inducible nitric oxide synthase (iNOS) and cyclooxygenase-2 (COX-2), which increased with LPS treatment, were decreased by 6-MC treatment. Mechanistic studies revealed that 6-MC reduced the phosphorylation of the mitogen-activated protein kinase (MAPK) family and IκBα in the MAPK and nuclear factor-kappa B (NF-κB) pathways, respectively. These results suggest that 6-MC is a potential therapeutic agent for inflammatory diseases that inhibits inflammation via the MAPK and NF-κB pathways.

## 1. Introduction

The inflammatory reaction is an essential defense mechanism that protects the body during pathogen invasion or tissue damage and can be described as an acute or chronic inflammatory reaction according to the action and period of occurrence [1,2]. In the acute inflammatory reaction, as macrophages are activated by antigens, pro-inflammatory cytokines (interleukin-1β (IL-1β), interleukin-6 (IL-6), tumor necrosis factor-α (TNF-α)), and inflammatory mediators (nitric oxide (NO), prostaglandin E_2_ (PGE_2_)) are secreted to remove foreign substances and are terminated through the process of tissue regeneration [3,4,5,6]. Symptoms during the inflammatory reaction include fever, swelling, and pain [7]. In this inflammatory response, macrophages are involved in host defense and homeostasis, and are activated by bacterial lipopolysaccharide (LPS), a cell wall component of Gram-negative bacteria, to express several inflammatory mediators. In the inflammatory response, macrophages receive stimuli through the surface receptor Toll-like receptor 4 (TLR4) [8,9]. This stimulus increases the production of pro-inflammatory cytokines (IL-1β, IL-6, and TNF-α) and inflammatory mediators (NO and PGE_2_) through the activation of phosphorylation of transcription factors nuclear factor-kappa B (NF-κB) and mitogen-activated protein kinase (MAPK). Thus, these inflammatory mediators can kill bacteria or remove tumors and eventually contribute to the body’s defenses [10,11,12,13,14,15,16,17,18,19,20,21]. However, it has been reported that pathological overexpression of these pro-inflammatory cytokines and inflammatory mediate factors may cause genetic mutations or tissue and nerve damage, resulting in fatal consequences for the body [22]. Recently, the overproduction of inflammatory mediators due to environmental pollution or increased stress has led to chronic inflammation or immune hypersensitivity reactions [23]. As a result, the incidence of several diseases such as rheumatoid arthritis, cancer, and inflammatory bowel disease is increasing [24,25,26,27,28,29,30]. Therefore, the regulation of the inflammatory response is very important for the maintenance of the living body, and the anti-inflammatory effects can be confirmed through the inhibitory effects on the production of inflammatory mediators such as NO, PGE_2_, and cytokines generated from the inflammatory reaction. Coumarin is synthesized from shikimic acid pathways in bacteria, fungi, and plants and its derivatives include umbelliferone, esculetin, herniarin, psoralen, and imperatorin. Recent studies have reported that these coumarin derivatives have biological activities, including antioxidant, anti-inflammatory, antibacterial, antiviral, and anti-cancer functions. In addition, various substituents in the coumarin skeleton may exhibit different biological activities because of their structural differences, which may increase or decrease their effects [31,32,33,34,35,36].

During our screening process to find functional substances of skin inflammation and melanogenesis from coumarin and its derivatives, it was observed that several coumarin derivatives have bioactivities on inflammation and melanogenesis. Based on these results, we reported that 6,7-dihydroxy-4-methylcoumarin inhibited inflammation via MAPK signaling pathways [37]. In addition, we identified that 7,8-dimethoxycoumarin attenuates inflammation via NF-κB and MAPK signaling pathways [38]. Furthermore, it was reported that 8-methoxycoumarin increased melanogenesis via MAPK pathways [39]. As an extension of this study, we screened several coumarins, which have similar structure, to identify the structural features involved in the bioactivities of this class of molecules.

**Figure 1:**
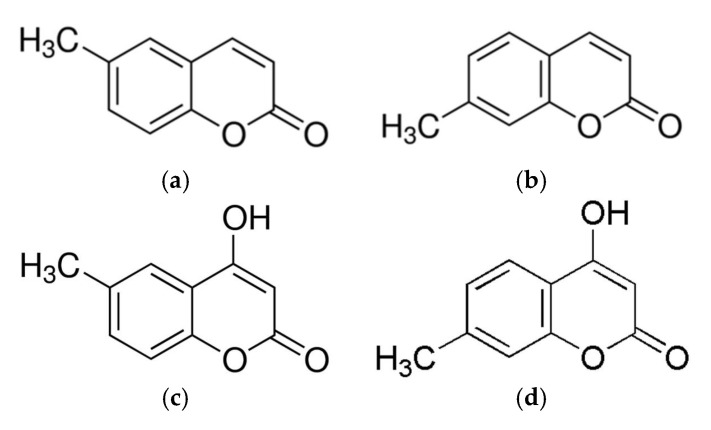
The chemical structure of (**a**) 6-methylcoumarin (6-MC), (**b**) 7-methylcoumarin (7-MC), (**c**) 4-hydroxy-6-methylcoumarin (4H-6-MC), and (**d**) 4-hydroxy-7-methylcoumarin (4H-7-MC).

In this study, we observed that among the compounds screened, four coumarin derivatives, (a) 6-methylcoumarin (6-MC), (b) 7-methylcoumarin (7-MC), (c) 4-hydroxy-6-methylcoumarin (4H-6-MC), and (d) 4-hydroxy-7-methylcoumarin (4H-7-MC), which have similar structures (Figure 1), exhibit distinct potency derived of their structural differences. In addition, the anti-inflammatory potential of each coumarin was evaluated based on their capacity to reduce cellular NO levels, the most promising coumarin being selected, and a mechanistic study conducted.

## 2. Results

### 2.1. Effect of Coumarin Derivatives on the Proliferation and NO Production of RAW 264.7 Cells

**Figure 2:**
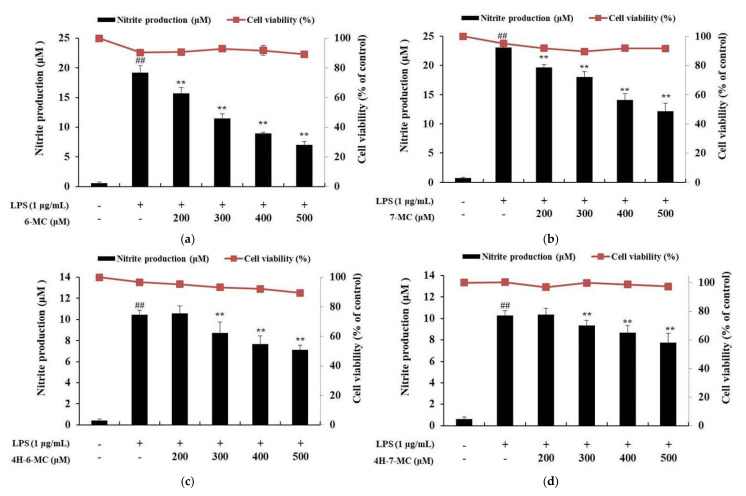
Effect of coumarin derivatives on nitric oxide production in LPS-stimulated RAW 264.7 cells. The cells were stimulated with 1 μg/mL of LPS only or with LPS plus various concentrations of coumarin derivatives for 24 h. Cell viability and NO production of LPS-induced RAW 264.7 cells subjected to (**a**) 6-methylcoumarin (6-MC), (**b**) 7-methylcoumarin (7-MC), (**c**) 4-hydroxy-6-methylcoumarin (4H-6-MC), and (**d**) 4-hydroxy-7-methylcoumarin (4H-7-MC) treatment was measured using MTT and Griess reagents, respectively. The results are presented as the mean ± SD from three independent experiments. ## *p* < 0.01 versus untreated control group. ** *p* < 0.01 versus LPS alone.

To investigate the cytotoxicity of coumarin derivatives against RAW 264.7 cells, cell viability was investigated using the 3-(4,5-dimethylthiazol-2-yl)-2,5-diphenyltetrazolium bromide (MTT) assay. The cells were treated with coumarin derivatives (200, 300, 400, and 500 μM) and LPS (1 μg/mL) and incubated for 24 h to measure absorbance. As a result, there were no significant differences between the untreated control and coumarin derivatives-treated group in RAW 264.7 cells (Figure 2). Therefore, coumarin derivatives were considered to have no cytotoxicity at the treated concentrations, and further studies were conducted using the following concentrations: 200, 300, 400, and 500 μM.

To examine the effect of coumarin derivatives on NO production, cells were pretreated with each coumarin derivative (200, 300, 400, and 500 μM) for 1 h and then stimulated for 24 h with LPS (1 μg/mL). The cells treated with LPS alone markedly induced NO production compared to the untreated control cells. However, NO production was significantly decreased by coumarin treatment in a concentration-dependent manner. In particular, 500 μM, which was the highest concentration tested, decreased the NO production level by 63.5% for 6-MC, 47.3% for 7-MC, 32.1% for 4H-6-MC, and 26.4% for 4H-7-MC, compared with the LPS alone-treated group (Figure 2a–d, ** *p* < 0.01).

### 2.2. Effect of Coumarin Derivatives on Proliferation and Melanin Contents of B16F10 Cells

**Figure 3:**
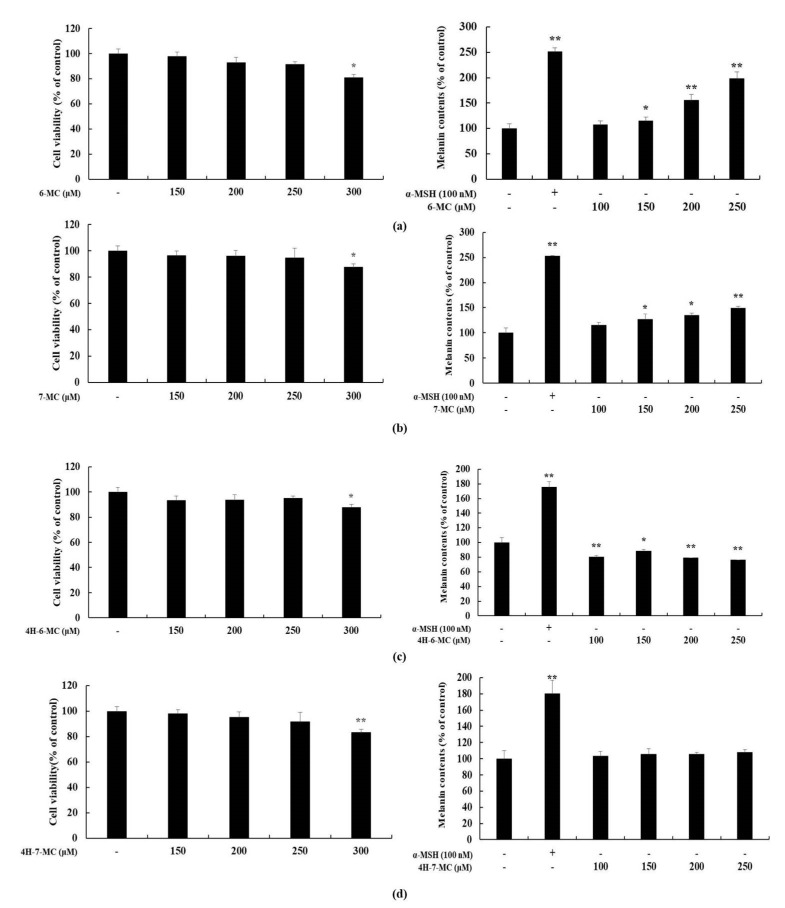
Effect of coumarin derivatives on proliferation and melanin contents in B16F10 cells. The cells were treated with various concentrations of coumarin derivatives for 72 h. α-MSH was used as the positive control. Proliferation and melanin contents of cells subjected to (**a**) 6-methylcoumarin (6-MC), (**b**) 7-methylcoumarin (7-MC), (**c**) 4-hydroxy-6-methylcoumarin (4H-6-MC), and (**d**) 4-hydroxy-7-methylcoumarin (4H-7-MC) treatment were expressed as percentages compared to the untreated control cells. The results are presented as the mean ± SD from three independent experiments. * *p* < 0.05, ** *p* < 0.01 versus untreated control cells.

Several studies have showed that the substances with anti-inflammatory activity are also involved in melanogenesis [21,38,39,40,41,42]. Therefore, to check whether these coumarin derivatives also affect the melanogenesis, MTT assay and melanin contents test were performed using B16F10 murine melanoma cells. The cells were treated with coumarin derivatives (150, 200, 250, and 300 μM) and incubated for 72 h to measure absorbance. α-melanocyte-stimulating hormone (MSH) (100 nM) was used as the positive control. The MTT assay result showed that there were no significant differences between the untreated control and coumarin derivatives-treated group at indicated concentrations (150, 200, and 250 μM) in B16F10 cells. Therefore, coumarin derivatives were considered to have no cytotoxicity at concentrations under 250 μM. The melanin content test was conducted using the following concentrations: 100, 150, 200, and 250 μM (Figure 3). The result of melanin contents test showed that, the cells treated with 6-MC showed an increase in melanin content compared to that in the untreated control cells (Figure 3a). However, there were no significant differences between the untreated control cells and other coumarin derivative-treated groups (Figure 3b–d). Based on NO production and melanin contents results, it can be confirmed that the bioactivity of 6-MC is better than that of other coumarin derivatives. Therefore, further experiments were performed to investigate the anti-inflammatory effects of 6-MC.

### 2.3. Effect of 6-MC on PGE_2_ and Pro-Inflammatory Cytokines Expression

**Figure 4:**
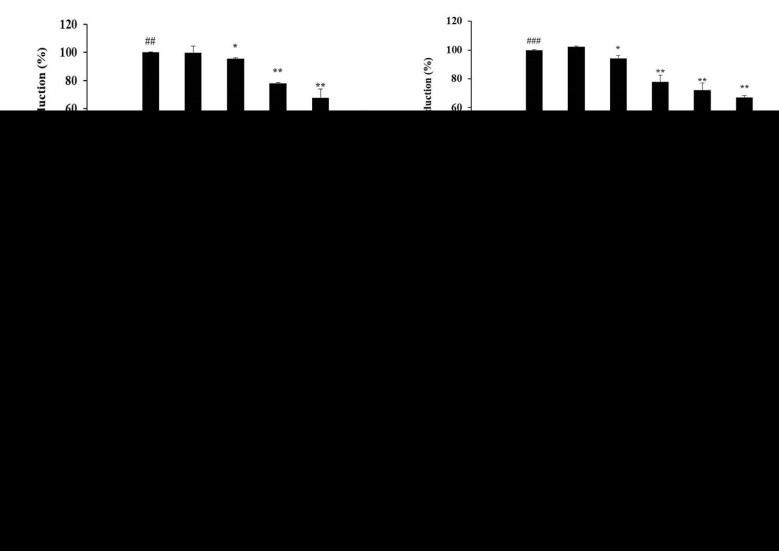
Effect of 6-MC on the production of PGE_2_ and pro-inflammatory cytokines in LPS-induced RAW 264.7 cells. Cells were pretreated with 6-MC for 1 h and subsequently stimulated for 24 h with LPS. (**a**) PGE_2_, (**b**) TNF-α, (**c**) IL-6, and (**d**) IL-1β production was determined by ELISA. The results are presented as the mean ± SD from three independent experiments. ## *p* < 0.01 vs. untreated control group. * *p* < 0.05, ** *p* < 0.01 vs. group treated with LPS alone.

To examine the effect of 6-MC on the expression of PGE_2_ and pro-inflammatory cytokines such as TNF-α, IL-6, and IL-1β, LPS-stimulated RAW 264.7 cells were treated with various concentrations. The expression levels of cytokines were measured using ELISA kits. The cells treated with LPS alone showed a marked increase in inflammatory cytokine levels compared to those in the untreated control cells. However, the levels of PGE_2_, TNF-α, IL-6, and IL-1β were significantly decreased in 6-MC-treated cells in a concentration-dependent manner. In particular, 500 μM, which was the highest treatment concentration, markedly decreased PGE_2_, TNF-α, IL-6, and IL-1β expression levels by 53.2%, 32.8%, 73.1%, and 80.6%, respectively, compared with the LPS alone-stimulated group (Figure 4a–d, ** *p* < 0.01).

### 2.4. Measurement of iNOS and COX-2 Protein Expression

**Figure 5:**
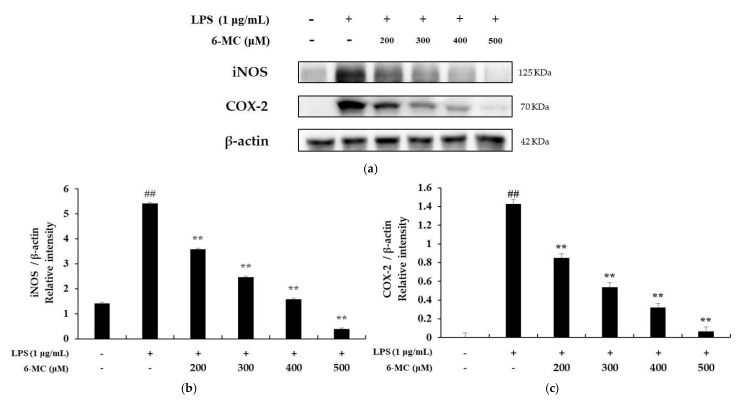
Effect of 6-MC on the protein expression level of iNOS and COX-2 in LPS-induced RAW 264.7 cells. (**a**) Result of Western blotting, and protein levels of (**b**) iNOS and (**c**) COX-2. Lysates were prepared from cells pretreated with 6-MC (200, 300, 400, and 500 μM) for 1 h and then treated with LPS (1 μg/mL) for 18 h. β-actin was used as a loading control. Total cellular proteins were separated using SDS-PAGE, transferred to PVDF membranes, and detected using specific antibodies against iNOS, COX-2, and β-actin. The results in the graphs are presented as the mean ± SD from three independent measurements using the ImageJ program. ## *p* < 0.01 vs. untreated control group. ** *p* < 0.01 versus group treated with LPS alone.

Recent studies have reported that NO and PGE_2_ production is regulated by iNOS and COX-2 protein expression [43,44]. Therefore, the expression of iNOS and COX-2 was confirmed by Western blotting to determine whether the expression of inflammatory mediators is related to the expression of iNOS and COX-2. As shown in Figure 4a, iNOS expression was increased 3.85-fold in cells treated with LPS alone compared to that in untreated control cells. However, it was decreased by 33.8%, 54.52%, 70.79%, and 92.79% at 6-MC treatment concentrations of 200, 300, 400, and 500 μM, respectively (Figure 5a, ** *p* < 0.01). In addition, COX-2 expression was increased by 35.7-fold in the LPS alone-treated group compared with untreated control cells, and it was decreased by 40.8%, 62.7%, 78.2%, and 95.78%, respectively (Figure 5b, ** *p* < 0.01), in the presence of 6-MC (200, 300, 400, and 500 μM) (Figure 4b). Therefore, the results suggest that 6-MC suppresses the production of NO and PGE_2_, which are inflammatory mediators, through inhibition of iNOS and COX-2 protein expression, respectively.

### 2.5. Effect of 6-MC on the Phosphorylation of MAPK

**Figure 6:**
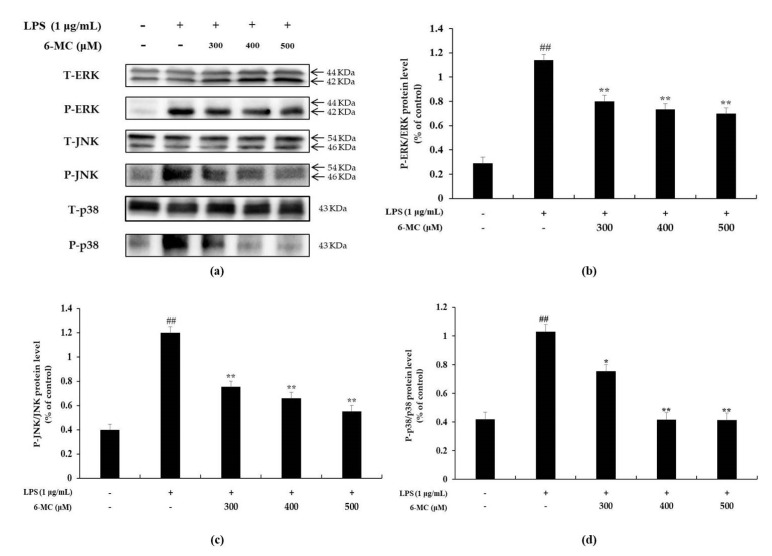
Effect of 6-MC on phosphorylation level of MAPK in LPS-induced RAW 264.7 cells. (**a**) Result of Western blotting, and protein levels of (**b**) P-ERK/ERK, (**c**) P-JNK/JNK, and (**d**) P-p38/p38. Lysates were prepared from cells pretreated with 6-MC (300, 400, and 500 μM) for 1 h and then treated with LPS (1 μg/mL) for 15 min. β-actin was used as a loading control. Total cellular proteins were separated using SDS-PAGE, transferred to PVDF membranes, and detected using specific antibodies against phospho-ERK, T-ERK, phospho-JNK, T-JNK, phospho-p38, and T-p38. P: phosphorylated, T: total. The results in the graphs are presented as the mean ± SD from three independent measurements using the ImageJ program. ## *p* < 0.01 vs. untreated control group. * *p* < 0.05, ** *p* < 0.01 versus group treated with LPS alone.

It has been reported that LPS stimulates TLR4 on the surface of macrophages to induce phosphorylation of MAPK (extracellular signal regulated kinase; ERK, c-Jun N-terminal kinase; JNK, p38) to activate a cell signaling pathway and increase the expression of various inflammatory cytokines [11,12,13]. Therefore, Western blotting was performed to confirm whether the inhibitory effect of 6-MC on inflammatory cytokine expression was due to the inhibition of MAPK phosphorylation. As shown in Figure 6, there was significant phosphorylation of ERK (4.1-fold), JNK (3.1-fold), and p38 (2.5-fold) in cells treated with LPS alone compared to untreated control cells. It was also confirmed that LPS-stimulated phosphorylation of ERK, JNK, and p38 was attenuated by 6-MC treatment in a concentration-dependent manner. In particular, 500 μM, which was the highest treatment concentration, markedly decreased ERK, JNK, and p38 phosphorylation levels by 38.9%, 53.8%, and 59.7%, respectively, compared with the LPS alone-stimulated group (Figure 6b–d, ** *p* < 0.01). Thus, the results suggest that 6-MC suppresses inflammatory cytokine expression by blocking MAPK phosphorylation.

### 2.6. Effect of 6-MC on the NF-κB Signaling Pathway

**Figure 7:**
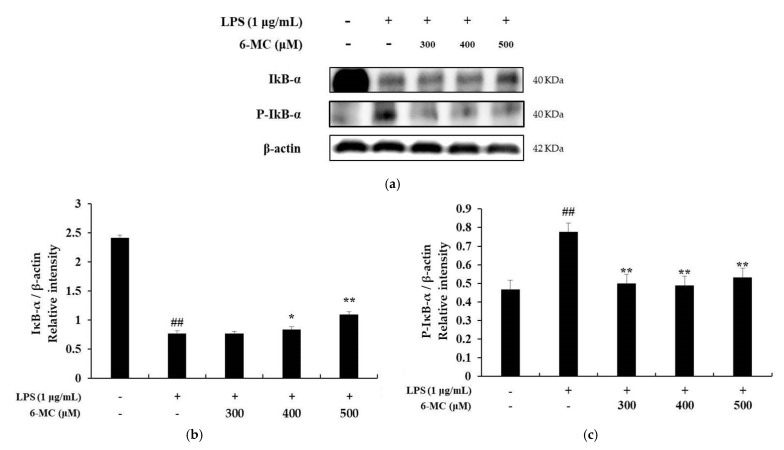
Effect of 6-MC on the protein expression level of P-IκB-α and IκB-α in LPS-induced RAW 264.7 cells. (**a**) Result of Western blotting, and protein level of (**b**) IκB-α and (**c**) P-IκB-α. Lysates were prepared from cells pretreated with 6-MC (300, 400, and 500 μM) for 1 h and then treated with LPS (1 μg/mL) for 20 min. β-actin was used as a loading control. Total cellular proteins were separated using SDS-PAGE, transferred to PVDF membranes, and detected using specific antibodies against phospho-IκB-α, IκB-α, and β-actin. The results in the graphs are presented as the mean ± SD from three independent measurements using the ImageJ program. ## *p* < 0.01 vs. untreated control group. * *p* < 0.05, ** *p* < 0.01 versus group treated with LPS alone.

**Figure 8:**
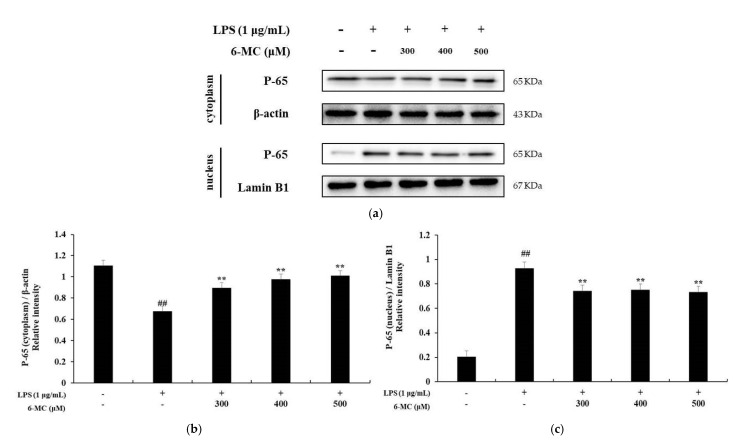
Effect of 6-MC on the protein expression level of NF-κB (P-65; cytoplasm) and (P-65; nucleus) in LPS-induced RAW 264.7 cells. (**a**) Result of Western blotting, and protein level of (**b**) P-65 (cytoplasm) and (**c**) P-65 (nucleus). Lysates were prepared from cells pretreated with 6-MC (300, 400, and 500 μM) for 1 h and then treated with LPS (1 μg/mL) for 15 min. β-actin and Lamin B1 were used as a loading control. Total cellular proteins were separated using SDS-PAGE, transferred to PVDF membranes, and detected using specific antibodies against NF-κB/P-65, Lamin B1, and β-actin. The results in the graphs are presented as the mean ± SD from three independent measurements using the ImageJ program. ## *p* < 0.01 vs. untreated control group. ** *p* < 0.01 versus group treated with LPS alone.

A previous study reported that in LPS-stimulated macrophages, IκB-α is phosphorylated by IκB-α kinase and subsequently ubiquitinated. Consequently, IκB-α is released from the NF-κB complex and undergoes degradation. In addition, phosphorylated NF-κB (p50 and p65) is translocated to the nucleus, increasing the production of inflammatory cytokines and proteins, including iNOS and COX-2 [45]. To investigate the molecular mechanism of 6-MC-mediated inhibition of inflammatory cytokine expression, NF-κB pathway activity was investigated using a Western blot assay. As shown in Figure 7, LPS stimulation of cells decreased IκB-α expression. However, it was significantly increased by 6-MC (300, 400, 500 μM) treatment, with a 43.4% increase at 500 μM (Figure 7b, ** *p* < 0.01). Phosphorylation of IκB-α was markedly induced by LPS treatment, but it was decreased by 6-MC treatment, with a 35.8% decrease at 500 μM (Figure 7c, ** *p* < 0.01). Next, we confirmed the translocation of NF-κB/p65 from the cytoplasm to the nucleus. In the cytoplasm, p65 expression in cells treated with LPS was decreased compared to that in untreated control cells, while treatment with 6-MC significantly prevented the inhibition of p65 expression in a concentration-dependent manner. In the nucleus, the expression of p65 was increased in cells treated with LPS compared to untreated control cells, but it was inhibited by 6-MC treatment (Figure 8). These results suggest that 6-MC prevents IκB-α degradation and NF-κB nuclear translocation, thereby impeding inflammation.

## 3. Discussion

Various coumarins and their derivatives exist in natural products and have been extensively studied for their biological activities, including antioxidant, anti-inflammatory, antibacterial, antiviral, and anti-cancer functions [34,35,36]. In addition, various substituents in the coumarin skeleton may exhibit different biological activities owing to their structural differences [31]. Therefore, the comparison of biological activity based on structural differences would be useful for predicting effective functional substances. In this study, we selected coumarin derivatives with similar structures and compared their NO production inhibitory activities. By comparing 6-methylcoumarin and 7-methylcoumarin, we found that the NO inhibitory activity of 6-methylcoumarin was better. Interestingly, when a hydroxyl group was attached to the 4-position of 6-methylcoumarin (4H-6-MC) and 7-methylcoumarin (4H-7-MC), the NO inhibitory activity of coumarin was decreased (Figure 1 and Figure 2). To check whether this phenomenon also affects the activity of other cells, a melanin content test was performed using B16F10 cells. As a result, it was confirmed that melanin production increased in a concentration-dependent manner in 6-MC, which had the best NO production inhibitory activity (Figure 3a), and it was also confirmed that melanin content slightly increased in 7-MC (Figure 3b). However, it was confirmed that when a hydroxyl group was attached to the 4-position of 6-methylcoumarin (4H-6-MC) and 7-methylcoumarin (4H-7-MC), it is slightly decreased in melanin contents (Figure 3c) or it did not affect melanogenesis (Figure 3d). Therefore, it is considered that the biological activity of coumarin is affected by the hydroxyl group at position 4 in the coumarin skeleton. Based on these results, we conducted an anti-inflammatory study using 6-MC, which has the best biological activity in NO inhibition and melanin activation.

Recent studies reported that coumarin and its derivatives related to anti-inflammatory activities. It has reported that auraptene, a natural bioactive monoterpene coumarin ether, inhibits inflammation via the p38 MAPK signaling pathway in RAW 264.7 cells [46]. In addition, it was confirmed that esculin, a coumarin glucoside, exhibited anti-inflammatory activities in vivo and regulated TNF-α and IL-6 production in LPS-stimulated mouse peritoneal macrophages in vitro through the MAPK pathway [47]. Furthermore, it was studied that various coumarin derivatives such as 7,8-dimethoxycoumarin, 6,7-dihydroxy-4-methylcoumarin, and 4-hydroxy-7-methoxycoumarin have anti-inflammatory activity via the MAPK and NF-κB signaling pathways [37,38,48]. Therefore, in this study, we focused on the anti-inflammatory activity of 6-MC and its mechanisms and observed that 6-MC attenuates inflammation via the MAPK and NF-κB signaling pathway-dependent inflammation-mediated factors and pro-inflammatory cytokines downregulation in RAW 264.7 cells.

To determine the effect of 6-MC on inflammation, NO, PGE_2_, TNF-α, IL-6, and IL-1β levels were measured using Griess reagent and an ELISA kit. The results showed that the levels of inflammation-related cytokines and mediators were reduced by 6-MC treatment in a concentration-dependent manner (Figure 2 and Figure 4). In addition, to confirm whether NO and PGE_2_ levels were regulated by iNOS and COX-2 expression, a Western blot assay was performed to measure protein expression levels. As a result, protein expression was markedly induced by LPS treatment, but was significantly attenuated by 6-MC treatment in a dose-dependent manner (Figure 5). Based on this result, 6-MC reduced inflammation-mediated factors via downregulation of iNOS and COX-2 protein expression. To confirm whether 6-MC is related to the MAPK and NF-κB pathways, which are inflammation-related signaling pathways, a Western blot experiment was performed. It has been reported that the phosphorylation of MAPK (ERK, JNK, and p38) is closely associated with the expression of inflammatory cytokines. ERK in the ERK signaling pathway is phosphorylated by various stimulating factors, whereas p38 and JNK constitute a stress response pathway and are phosphorylated by cellular stress induced by factors such as inflammatory cytokines [11,12,13]. The result showed that phosphorylation of the MAPK family (ERK, JNK, p38) was reduced by 6-MC treatment (Figure 6). Therefore, 6-MC suppresses the MAPK signaling pathway by inhibiting phosphorylation of the MAPK family. A previous study has reported that in LPS-stimulated RAW 264.7 cells, IκB-α is phosphorylated and released from the NF-κB complex; consequently, NF-κB (p50 and p65) is translocated from the cytoplasm to the nucleus, upregulating the production of inflammatory cytokines [45]. In our study, LPS treatment decreased IκB-α expression, which was significantly increased by 6-MC treatment. Additionally, phosphorylation of IκB-α was induced by LPS treatment, whereas it was significantly decreased by 6-MC treatment in a dose-dependent manner (Figure 7). Moreover, the cells treated with 6-MC were significantly protected from the inhibition of p65 expression in the cytoplasm, and the expression of p65 was decreased by 6-MC treatment in the nucleus (Figure 8). These results suggest that 6-MC suppresses the activity of the NF-κB signaling pathway by inhibiting the degradation of IκB-α in the cytoplasm and preventing NF-κB translocation to the nucleus. The results of mechanistic studies suggest that 6-MC inhibits inflammatory cytokine expression by downregulating the MAPK and NF-κB signaling pathways.

**Figure 9:**
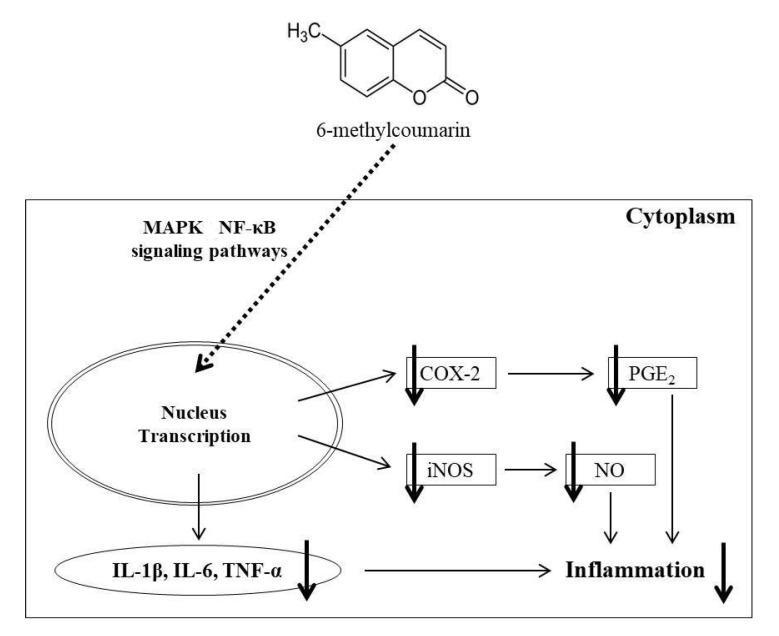
6-MC induces anti-inflammatory effects by inhibiting the production of inflammatory mediators (NO and PGE_2_) and pro-inflammatory cytokines (IL-1β, IL-6, and TNF-α) via MAPK and NF-κB signaling pathways in the cells treated with LPS.

In summary, this study is the first to evaluate the inhibitory effect of 6-MC on inflammation in RAW 264.7 macrophages. Moreover, 6-MC was shown to exert anti-inflammatory effects in cells by inhibiting the production of inflammatory mediators and pro-inflammatory cytokines via the inhibition of MAPK phosphorylation and protection of IκB-α degradation in the cells treated with LPS (Figure 9). These findings suggest that 6-MC is a potential therapeutic agent for inflammatory diseases, such as dermatitis, arthritis, cardiovascular disease, and cancer.

## 4. Materials and Methods

### 4.1. Chemicals and Reagents

6-Methylcoumarin (6-MC), 7-methylcoumarin (7-MC), 4-hydroxy-6-methylcoumarin (4H-6-MC), 4-hydroxy-7-methylcoumarin (4H-7-MC), dimethyl sulfoxide (DMSO), radioimmunoprecipitation assay (RIPA) buffer, Griess reagent, sodium nitrite, protease inhibitor cocktail, and phosphate buffered saline (PBS) were obtained from Sigma-Aldrich (St. Louis, MO, USA). Lipopolysaccharides derived from *Escherichia coli* (LPS) and 3-(4,5-dimethylthiazol-2-yl)-2,5-diphenyltetrazolium bromide (MTT) were obtained from VWR (Radnor, PA, USA). Dulbecco’s modified Eagle’s medium (DMEM), fetal bovine serum (FBS), penicillin/streptomycin, and nuclear and cytoplasmic extraction reagents (NCER) were obtained from Thermo Fisher Scientific (Waltham, MA, USA). Tris-buffered saline (TBS) and enhanced chemiluminescence (ECL) kits were obtained from Biosesang (Seongnam, Gyeonggi-do, Korea). The following antibodies were used: β-actin, anti-iNOS, anti-COX-2, T-IκBα, p-IκBα, T-ERK, p-pERK, T-JNK, p-JNK, T-p38, and p-p38. They were obtained from BD Biosciences (San Diego, CA, USA). The interleukin-6 (IL-6), interleukin-1β (IL-1β), prostaglandin E_2_ (PGE_2_), and tumor necrosis factor (TNF-α) ELISA kits were obtained from R&D Systems Inc. (St, Louis, MO, USA). The protein levels were quantified and graphed using the ImageJ program (NIH, Bethesda, MD, USA). All reagents used above were of analytical grade.

### 4.2. Cell Culture

RAW 264.7 mouse macrophage cells were obtained from the Korean Cell Bank (Seoul, Korea). B16F10 mouse melanoma cells were purchased from the Global BioResource Center (ATCC). RAW 264.7 and B16F10 cells were subcultured at intervals of 2 and 4 days, respectively, using DMEM containing 10% FBS and 1% penicillin/streptomycin. The cells were maintained in 5% CO_2_ at 37 °C.

### 4.3. Measurement of Cell Viability

Cell viability was determined using the MTT assay. RAW 264.7 murine macrophage cells were cultured at 1.5 × 10^5^ cells/well in 24-well plates for 24 h. The cells were then treated with various concentrations of 6-MC (200, 300, 400, and 500 µM) for 24 h. B16F10 cells were cultured at 1.5 × 10^4^ cells/well in 24-well plates for 24 h. After that, the cells were treated with various concentrations of 6-MC (50, 100, 150, 200, 250, and 300 µM) for 72 h. Next, MTT solution (0.2 mg/mL) was added to the medium for 4 h, the medium was removed, and the formazan crystals in each well were dissolved in DMSO to determine cell viability. Absorbance was measured at 570 nm with a spectrophotometer, and the percentage of cells exhibiting cell viability compared to the control was determined.

### 4.4. Measurement of NO Production

The NO production accumulated in the cell culture was measured in the form of nitrite using Griess reagent. RAW 264.7 mouse cells were plated at 1.5 × 10^5^ cells/well in 24-well plates. The cells were pretreated with various concentrations of samples (200, 300, 400, and 500 µM) for 1 h, and then treated with LPS (1 μg/mL) for 24 h. Thereafter, the cell supernatant and Griess reagent were mixed in a 100 µL reaction mixture (50 µL of cell supernatant and 50 µL of Griess reagent) for 20 min. Absorbance was measured at 540 nm using a spectrophotometer. A standard curve was created using sodium nitrite to quantify the amount of NO produced in the sample.

### 4.5. Measurement of Intracellular Melanin Content

B16F10 melanoma cells were seeded in a 60 mm dish at 6.0 × 10^4^ cells/dish and incubated for 24 h. The cells were then treated with coumarin derivatives (100, 150, 200, and 250 µM) and α-MSH (100 nM) for 72 h. α-MSH served as the positive control. After the supernatant was removed, the cells were washed twice with phosphate buffered saline (PBS), and the cell pellets were collected and dissolved in 1 N NaOH for 1 h at 70 °C. Each cell lysate was transferred to a 96-well plate, and the absorbance of each well was determined at 540 nm using a spectrophotometer.

### 4.6. Measurement of Cytokines

RAW 264.7 mouse cells were plated at 1.5 × 10^5^ cells/well in 24-well plates. The cells were pretreated with various concentrations of samples (200, 300, 400, and 500 µM) for 1 h, and then treated with LPS (1 μg/mL) for 24 h. Supernatants were harvested, and the levels of PGE_2_ and pro-inflammatory cytokines IL-6, IL-1β, and TNF-α were measured using ELISA kits according to the manufacturer’s protocol.

### 4.7. Preparation of Nuclear and Cytosolic Extraction

Nuclear and cytosolic extracts were fractionated using a previously reported procedure [49]. Briefly, after washing once with PBS, the cells were harvested using trypsin-EDTA and then centrifuged at 500× *g* for 5 min. After that, the pellet was washed with PBS and centrifuged at 500× *g* for 3 min, after which the supernatant was removed. After adding 200 μL of cytoplasmic extraction reagent (CER)1 on ice, the mixture was vortexed for 15 s and allowed to react in a cold state for 10 min. After adding 11 μL of cytoplasmic extraction reagent (CER)2 on ice, the mixture was vortexed for 5 s and allowed to react in a cold state for 1 min. After vortexing for 5 s, centrifugation was performed at 16,000× *g* for 5 min. The supernatant was stored in an e-tube and used as a cytoplasmic protein. After adding 100 μL of nuclear extraction reagent (NER) to the remaining pellet on ice, it was vortexed for 15 s and reacted in a cold state for 10 min. This process was repeated four times. The samples were centrifuged at 16,000× *g* for 10 min, and the supernatant was transferred to an e-tube and used as a nuclear protein. The separated proteins were stored at −80 °C.

### 4.8. Western Blot Analysis

RAW 264.7 mouse cells were plated at a density of 7.0 × 10^5^ cells/dish in a 60-mm cell culture dish for 24 h. Cells were pretreated with various concentrations of 6-MC (200, 300, 400, 500 μM) for 1 h, treated with LPS (1 μg/mL) for the specified time with cold 1× PBS, and washed twice. After lysis for 20 min in RIPA lysis buffer (150 mM NaCl, 50 mM Tris-HCl [pH 7.5], 2 mM EDTA, 1% Triton X-100, 0.1% SDS, 10% NP40, and 1% protein inhibitor cocktail), the lysate was transferred to a microtube and centrifuged at −8 °C and 15,000 rpm for 20 min. A protein was obtained from a cell lysate extracted using the BCA Protein Assay Kit, which is a standard calibration curve for bovine serum albumin (BSA). The content was calculated quantitatively. Whole-cell lysates (20 μg) were separated by SDS-polyacrylamide gel electrophoresis on a 10% gel (SDS-PAGE) and electro-transferred to polyvinylidene fluoride (PVDF) membranes. The membranes were incubated for 2 h with 5% skim milk. Next, the membrane was incubated for 8 h with specific primary antibodies (1:2000). The membrane was washed six times with TTBS buffer every 10 min and incubated for 2 h at 27 °C with a peroxidase-conjugated secondary antibody (1:1000). Finally, the membrane was washed six times with TTBS buffer every 10 min, and the proteins were detected using an ECL kit.

### 4.9. Data Analysis

All experimental results are expressed as the mean ± standard deviation of three independent experiments. Statistical analysis was performed using the Student’s *t*-test. *p*-values < 0.05 (*) or 0.01 (**) were considered statistically significant.

## Data Availability

Not applicable.

## Sample Availability

Samples of the compounds are available from the authors.
